# The role of environmental efficiency and economic development in fertility: implications for public health and sustainability among OECD nations

**DOI:** 10.3389/fpubh.2025.1551413

**Published:** 2025-02-20

**Authors:** Renyan Mu, Fuang Zhang, Shidi He, Jingshu Zhang

**Affiliations:** ^1^School of Management, Wuhan University of Technology, Wuhan, China; ^2^Center for Product Innovation Management of Hubei Province, Wuhan, China

**Keywords:** fertility rate, air pollution, environmental efficiency, economic development, sustainability, DEA

## Abstract

**Introduction:**

As global economies rapidly develop, the interplay between environmental efficiency, economic development, and public health outcomes has gained significant attention. Air pollution and resource-intensive economic activities threaten both environmental sustainability and human health, including reproductive health and overall well-being.

**Methods:**

This study focuses on OECD member countries, using data from 1999 to 2021. An undesirable outputs-oriented DEA approach is employed to assess environmental efficiency across these countries. Baseline regression analysis is conducted to examine the relationship between environmental efficiency and fertility, while heterogeneity analysis explores the impact of industrial and energy consumption structures. Additionally, the moderating effect of economic development levels is investigated.

**Results:**

The baseline regression results indicate an inverted U-shaped relationship between environmental efficiency and fertility, where fertility initially declines as environmental efficiency increases, then rises after reaching a certain threshold. Heterogeneity analysis reveals that industrial and energy consumption structures significantly influence this relationship across different regions. Furthermore, economic development is found to be a reverse moderator: in countries with higher economic development levels, the relationship between environmental efficiency and fertility follows a significant U-shaped curve.

**Discussion:**

These findings highlight the necessity of integrating environmental policies with public health strategies. Improvements in environmental efficiency may reduce pollution-related health risks, indirectly supporting fertility recovery in advanced economies. By addressing the interaction between environmental efficiency, economic development, and fertility, this study provides evidence-based insights for designing policies that promote sustainable environmental health and equitable social outcomes.

## 1 Introduction

Public health is a critical pillar of sustainable development, directly influencing human well-being (1). Increasing health challenges globally are closely linked to environmental pollution, with air pollution, water contamination, and exposure to harmful chemicals posing significant threats to human health, while also placing additional burdens on public health systems, economic prosperity, and social stability (2–4). Environmental degradation, including pollution-related diseases such as chronic illnesses and respiratory conditions, particularly affects vulnerable populations. Furthermore, the three main objectives of sustainable development—economic prosperity, social stability, and ecological security—are intrinsically linked to public health outcomes, including fertility rates, which are essential to human sustainability (1, 5).

The impact of environmental pollution on reproductive health is particularly significant, directly influencing population dynamics and society’s long-term stability (6, 7). The decline in fertility rates has become a major issue for many developed countries, driven not only by social and economic factors but also by environmental influences, particularly pollution’s detrimental effects on reproductive health (8, 9). The decline in fertility leads to an aging population, increasing the strain on healthcare and social welfare systems (10). While economic development and rising living standards are generally associated with lower fertility rates, this relationship is not purely negative. An increasing body of research indicates that environmental pollution and exposure to harmful substances significantly affect reproductive health, underscoring the need for the integration of economic and ecological factors (11, 12). Over the past few decades, many Asian and European countries have experienced dramatic declines in fertility, and it is estimated that by 2030, nearly two-thirds of the global population will reside in countries with a fertility rate below 2.1 (9, 13). This trend highlights the urgent need to integrate public health strategies with sustainable development goals to ensure both environmental and human well-being.

Fertility has consistently been a central focus of demographic research, attracting significant interest in recent years from several fields such as economics and medicine. Cultural and structural theories are the two main categories of ideas that have been created in the economic literature to explain the fall in fertility (14). The former focuses on how fertility is restricted by the emergence as well as the dissemination of innovative ideas and technologies. The latter, which incorporates theories of demand and socioeconomics, emphasizes how couples are inspired to delay having children by shifting social and economic circumstances, such as growing salaries, increased educational attainment, and women’s growing engagement in the workforce. There appears to be a strong and consistent correlation between falling fertility and growth in the economy. More recent evidence, however, has been offered by some researchers based on international data, suggesting that the relationship between both shifts from negative to positive when economic development reaches a certain level (15, 16). The finding gives doubt to the commonly held belief that fertility and economic development are inversely correlated, triggering debates over the inverse J-shaped relationship between the two variables. The majority of the literature in the field of medicine examines pathological causes and how epidemiological research views them in terms of human reproductive capacity. Pathological factors that contribute to infertility affecting males and females include both external environmental causes and internal genetic factors. Environmental factors include a wide range of toxins, including heavy metal pollutants like arsenic (As), mercury (Hg), cadmium (Cd), and lead (Pb), as well as air pollutants like PM2.5, NO_2_, and SO_2_ (11, 17). All of these pollutants have the potential to severely compromise human reproduction. The hazards of air contamination on sperm motility and morphology have been proved in the literature. For example, Guven et al. revealed that sperm motility can be reduced by 15.49 to 22.1% when exposed to air pollution (or vehicle exhaust) (18). Furthermore, air pollution may affect the timing of female ovulation (19), raising the likelihood of a spontaneous miscarriage and the risk of stillbirths in pregnant women (20).

Intending to realize dynamic, healthy growth, the theory of sustainable development takes into account the economy, environment, and society as an integrated whole, highlighting the coordinated limits and combined consideration of diverse characteristics. To comprehend the relationship between the sustainability of the economy and the environment, the reduction in fertility rates—an essential issue affecting the continuation of human society—is explored from the standpoint of sustainable development. It serves to reveal the system’s “black box,” providing insights into how sustainable development goals will be realized.

Moreover, disagreements and divergent findings about specific topics in different domains continue to surround fertility research. Further supplementation is required since there is insufficient research exploring the factors impacting fertility rates from a multidisciplinary perspective. Therefore, this study explores the relationship between environmental efficiency and fertility rates during the process of economic development by combining environmental and economic factors into the same research framework while launching from the perspective of sustainable development. As previously stated, given the ongoing debate surrounding the inverse J-shaped relationship, we will analyze the role that the economic development level plays in further detail. The results of the study show an interrelationship between economic and environmental factors and their collective impact on fertility, establishing a theoretical framework for examining the relationship between environmental efficiency and fertility. We also present empirical evidence on the fertility rates and environmental performance of various countries’ economic growth.

The remaining sections are as follows. Section 2 elucidates the theoretical foundations and presents the research hypotheses. Section 3 outlines the methodology and data sources. Section 4 reports the results and conducts robustness tests and heterogeneity analyses. Section 5 discusses the moderating role of economic development. Section 6 summarizes the conclusions and puts forward several recommendations.

## 2 Theoretical analysis and research hypotheses

### 2.1 Exposure to pollutants and fertility

Air contamination poses a significant threat to human reproductive capacity. The prevalence of infertility, encompassing male infertility and female infertility, stands as a crucial factor influencing fertility rates. Approximately 8–12% of couples worldwide confront infertility, with 50% of occurrences caused by the male component (21). The widespread distribution of environmental pollutants is one of the main drivers of the rising incidence of male infertility globally (7). In the fields of toxicology and epidemiology, researchers explore the connection between environmental contaminants and biomarkers of childlessness. Some studies indicate that air pollution significantly impacts human fertility and sperm quality (6, 12). Similarly, research on the influence of air pollution on female reproduction has reached consistent conclusions. Air pollution adversely affects various aspects of the female reproductive process, including the morphologic changes of ovarian antral follicles (22, 23), early embryo development (24), and fetal growth and reproductive outcomes (25). Furthermore, employing data from 2010 census reports throughout China, Xue and Zhang connected contact with PM2.5 to fertility and discovered that there was a significant 2.0% decline in human fertility for every 10 mg/m^3^ increment of PM2.5 (11). Subsequently, they expanded this research, incorporating a temporal dimension to examine the correlation between the two factors based on spatial geography. Their research verified the biological rationality of a negative correlation between air quality and fertility by statistically confirming the association between the two (26).

### 2.2 Environmental efficiency and fertility

The sustainability of the world demands that countries achieve eco-friendly economic growth with reduced resource consumption. To ensure sustainable development and growth, it is necessary to give priority to environmental quality. Ecological footprint and CO_2_ emissions are the primary measures used in environmental quality literature (27, 28). Some studies have employed CO_2_ emissions to estimate hazards to the environment since they make up a significant portion of greenhouse gases (GHGs) and information is readily available (29–32). It should be noted that CO_2_ does not qualify as a pollutant in the air that can negatively impact human fertility. The synergistic nature of control measures, however, as well as the co-emission features of carbon emissions and air contamination have been verified (33–35). Therefore, we use carbon emission efficiency as a proxy indicator for environmental efficiency in economic growth and employ carbon emissions as an equivalent for environmental pollutants produced in economic production activities.

In the majority of previous studies on CO_2_ and fertility (36–40), fertility rates were only taken into account as a secondary factor influencing population growth, which led to the consideration of restrictive population policies as a potential way of mitigating climate change. In addition, population momentum restricts the impact of fertility on population size. Put another way, this demographic factor limits potential variations in population size, hence, even if fertility alters significantly, the change in population size is likely to be tiny (41). Consequently, little research has been conducted to investigate the direct connection between CO_2_ and fertility. In contrast to these studies, our method focuses on the direct effects on fertility. According to production theory, both desirable and undesirable outputs, or pollutants, are produced during the same manufacturing procedure (42). We incorporate CO_2_ into the research framework, using it as a proxy for undesirable outputs in the same production process to measure eco-friendly economic growth, based on the synergistic effects of reducing emissions of greenhouse gases and air pollution.

This study aims to explore the relationship between fertility rates and environmental efficiency within the context of economic growth and sustainable development. Previous research has suggested that environmental performance, including pollutant emissions per unit of economic growth, can have significant implications for fertility rates. The Environmental Kuznets Curve (EKC) hypothesis, for instance, posits an inverted U-shaped relationship between economic development and environmental degradation, implying that early stages of economic growth may worsen environmental conditions, while later stages may lead to improvements (43, 44). Studies have also highlighted the complex relationship between environmental pollution and fertility, with some suggesting that pollution negatively affects fertility rates by increasing infertility (6, 12), while others argue that economic growth and advancements in healthcare may partially counterbalance these negative effects (14, 16). In line with these perspectives, we hypothesize that the relationship between environmental efficiency and fertility is non-linear. At lower levels of environmental efficiency, pollution may significantly harm fertility, while at higher levels, improvements in environmental quality may lead to more favorable fertility outcomes. Therefore, we propose the following hypothesis:

*H1*: A nonlinear relationship might exist between fertility rate and environmental efficiency.

## 3 Methodology and data

To explore the relationship between environmental efficiency and fertility, we first evaluate each country’s carbon emission efficiency as a measure of its environmental efficiency in terms of economic growth, employing the environmental DEA technique with an undesirable output-oriented DEA model. Subsequently, we ran a fixed effects regression on the two variables.

### 3.1 Measurement of environmental efficiency

DEA has been acknowledged as an important approach that is better suitable for evaluating the performance of Decision-making Units (DMUs). According to production theory, a process can produce both desirable and undesirable outputs, which are also referred to as contaminants. Such procedures can be modeled in DEA by applying the weak disposability reference technique, which Färe et al. offered (45). When the production method is established, environmental performance can be assessed by employing the Shephard distance function or the directional distance function (42). Thus, this study carries out the DEA-oriented base model of undesirable outputs, as emphasized by Tyteca (46, 47). The study used data from the 38 OECD countries from 1999 to 2021.

This study takes three inputs, one desirable output, and one undesirable output according to previous research (48–50). They are capital, labor, energy, GDP, and carbon dioxide emissions, respectively. Here, the input indicators are measured by the total capital formation (billion 1995 US$ in purchasing power parities), the number of employees, and the total consumption of primary fossil energy. The desirable output is measured by the actual GDP of each country (billion 1995 US$ in purchasing power parities), and the undesirable output is measured by the direct carbon dioxide emissions of each country. Data can be obtained from the OECD databases, the U.S. Energy Information Administration (EIA) databases, and the World Development Indicators (WDI) databases. The DEA-oriented base model of undesirable outputs is represented as Equation 1:


(1) $$\begin{array}{l} EE=\lambda^{*}=\min\lambda \\ s.t.\{\begin{array}{c} \overset{K}{\underset{k=1}{\sum }}z_{k}x_{nk}\leq x_{n0}\;n=1,2,\cdots ,N\\ \begin{array}{cc} \overset{K}{\underset{k=1}{\sum }}z_{k}y_{mk}\geq y_{m0} & m=1,2,\cdots ,M \end{array}\\ \begin{array}{cc} \overset{K}{\underset{k=1}{\sum }}z_{k}u_{jk}=\lambda u_{j0} & j=1,2,\cdots ,J \end{array}\\ \begin{array}{cc} z_{k}\geq 0 & k=1,2,\cdots ,K \end{array} \end{array} \end{array}$$

The DMU under evaluation is indicated by the subscript “0” in this instance, and the optimal efficiency value for this DMU—a composite standardized efficiency measure that may be utilized to assess environmental performance—is represented by $\lambda^{*}$ . It equals to 1, which indicates that the DMU is efficient, and vice versa, it signifies inefficiency. Here, inputs are denoted by *x*, desirable outputs by *y*, and undesirable outputs by *u*.

### 3.2 Variable explanations and data sources

Total Fertility Rate (*TFR*) is the dependent variable. In demography, the fertility rate is a widely used indicator that reflects the level of fertility and reproductive capacity. It is the proportion of live births to women who are of childbearing age. *TFR* is obtained by adding the fertility rates of various age groups, assuming that each age group has the same fertility rate. It can be applied to the comparison of populations and areas. Thus, the level of fertility is represented here by the *TFR*. The data for *TFR* is obtained from the World Bank’s WDI database.

Environmental Efficiency (*EE*) is the explanatory variable. We employ the *EE* determined by the DEA model to quantify the degree of environmental friendliness in the process of economic growth to investigate the aggregate effects of economic and environmental factors on fertility from a sustainable development viewpoint. It serves as a comprehensive factor that takes into account both environmental and economic aspects. The variables used to compute *EE*, including capital, labor, energy, GDP, and carbon dioxide emissions, are sourced from the OECD, EIA, and WDI databases.

Generally, higher infant mortality rates typically correspond to increased fertility rates based on the “preventive effect.” In the meantime, the main reason given for the drop in fertility rates is usually cited as urbanization, which is a structural shift in society. Also, drawing on previous empirical research, we ultimately settled on the infant mortality rate (*BDR*), urbanization rate (*UPR*), female education level (*FER*), and female labor force participation rate (*FLR*) as control variables. To calculate the infant mortality rate, one is required to identify the percentage of under-one-year-old deaths per 1,000 live births. The proportion of women in the 25–34 age group who accomplished higher education serves as a proxy for female education level. The percentage of working-age women who are employed is referred to as the female labor force participation rate, while the percentage of people who live in urban regions is defined as the urbanization rate. Data for these control variables are sourced from the WDI and OECD, databases.

### 3.3 Baseline regression model design

We successively estimate the following fixed-effects panel data model for OECD nations to investigate if environmental performance in terms of economic growth, as evaluated by the efficiency values derived from the DEA model previously mentioned, affects fertility:


(2) $$\ln TFR_{\mathrm{it}}=\alpha_{0}+\alpha_{1}EE_{\mathrm{it}}+\overset{n}{\underset{k=1}{\sum }}\theta_{k}X_{\mathrm{it}}+\mu_{i}+\delta_{t}+\varepsilon_{\mathrm{it}}$$

(3) $$\ln TFR_{\mathrm{it}}=\beta_{0}+\beta_{1}EE_{\mathrm{it}}+\beta_{2}EE_{\mathrm{it}}^{2}+\overset{n}{\underset{k=1}{\sum }}\theta_{k}X_{\mathrm{it}}+\mu_{i}+\delta_{t}+\varepsilon_{\mathrm{it}}$$

Here, *μ_i_* captures individual heterogeneity, *δ_t_* represents time effects, *ε_it_* represents error term and *X_it_* denotes a series of control variables. First, we test whether environmental efficiency affects fertility through Equation 2. Equation 3 adds the square of environmental efficiency (*EE_it_^2^*) to Equation 2. It can test if an increase in environmental efficiency may have a non-linear impact on the logarithm of fertility levels (*lnTFR_it_*), assuming the results of Equation 2 are significant.

The data from 38 OECD nations for the years 1999–2021 are chosen for empirical analysis in this research based on data availability. All data can be obtained from the EIA, WDI, and OECD databases. The missing data for a small number of observations (less than 1% of total data) was supplemented using linear interpolation. This approach ensures that the impact of missing values on the validity of the results is minimized. The variables’ descriptive statistics are given in Table 1.

**Table 1:** Descriptive statistics of variables.

Variable	Mean	Standard deviation	Minimum	Maximum
lnTFR	0.495	0.205	−0.211	1.135
EE	0.328	0.232	0.001	1.000
EE^2^	0.161	0.216	0.000	1.000
BDR	5.392	4.700	0.700	38.600
FER	41.220	14.640	7.354	76.490
FLR	59.640	10.910	22.320	81.090
UPR	76.500	11.070	50.730	98.120

## 4 Baseline results analysis

### 4.1 Unit root test

Before applying the panel regression model, unit root tests were conducted for each variable to avoid spurious regression. The LLC test was used to check stationarity, with the null hypothesis assuming a unit root. This test was chosen because it is suitable for panel data where all cross-sectional units share a common unit root process, which aligns with the characteristics of our dataset of OECD countries that exhibit similar economic and environmental dynamics (51). Although other tests, such as IPS and Fisher-type tests, could be used, the LLC test was preferred. The IPS test allows for different unit root processes across units, which is less suitable for our dataset, as we expect a uniform trend across the countries (52). Additionally, the LLC test provides a more robust and consistent method under the assumption of a shared unit root process. As shown in Table 2, the null hypothesis of the LLC test is rejected for each variable, suggesting that all variables are stationary and do not exhibit unit roots. This indicates that the variables used in the regression analysis are stable, which is crucial for ensuring valid and reliable results in subsequent econometric modeling.

**Table 2:** The regression results of the baseline model.

	(1)	(2)	(3)	(4)
*EE*	0.101^***^	0.571^***^	0.114^***^	0.503^***^
	(0.0230)	(0.0698)	(0.0212)	(0.0614)
*EE2*		−0.435^***^		−0.359^***^
		(0.0612)		(0.0533)
*UPR*			−0.014^***^	−0.013^***^
			(0.0014)	(0.0014)
*BDR*			0.003^**^	0.003^*^
			(0.0014)	(0.0014)
*FER*			0.003^***^	0.003^***^
			(0.0005)	(0.0005)
*FLR*			0.010^***^	0.010^***^
			(0.0010)	(0.0010)
N	874	874	874	874
adj. R^2^	0.085	0.138	0.324	0.359

Standard errors in parentheses, ^*^p < 0.1, ^**^p < 0.05, ^***^p < 0.01.

### 4.2 Regression results and analysis

Table 3 illustrates the specific values of the baseline regression model. The linear association between EE and fertility is independently examined in column (1). The results of introducing control variables are displayed in column (3), where the coefficients rise from 0.101 to 0.114 and are all significant within the 1% level. It suggests that EE and fertility have a beneficial association. The results in columns (2) and (4) further test our H1 based on Equation 2. The linear term (*EE*) has coefficients of 0.571 and 0.503, respectively, which are significant within the 1% level and occur in the same way as what is found in Equation 2. Simultaneously, consistent conclusions are drawn from the quadratic term’s regression coefficients in Equation 3. Both have a negative sign, are pointing in the opposite direction of the linear term, and are significant at the 1% level. Columns (2) and (4) offered significant findings, with turning points at 0.67 and 0.70, both falling inside the interval of variable value. It implies that EE and fertility have an inverse U-shaped bond, supporting the H1.

**Table 3:** The results of baseline regression and their robustness tests.

	(1)	(2)	(3)	(4)	(5)
*EE*	0.503^***^	0.689^***^		0.153^**^	
	(0.0614)	(0.143)		(0.0632)	
*L.EE*					0.465^***^
					(0.0617)
*EE^2^*	−0.359^***^	−0.792^***^		−0.125^**^	
	(0.0533)	(0.305)		(0.0549)	
*L.EE^2^*					−0.330^***^
					(0.0539)
*EE3*		0.281			
		(0.195)			
*EE_low*			0.205^***^		
			(0.0283)		
*EE_high*			−0.268^***^		
			(0.0826)		
*high*			0.0184		
			(0.0164)		
*Control*	Yes	Yes	Yes	Yes	Yes
*N*	874	874	874	874	836
adj. *R*^2^	0.359	0.360	0.350	0.501	0.358

Standard errors in parentheses, ^*^p < 0.1, ^**^p < 0.05, ^***^p < 0.01.

Previous studies commonly support the view that poor air quality is negatively correlated with reproductive health in parents, which can affect pregnancy outcomes and reduce fertility rates (6, 11, 26). Similarly, our findings suggest that improvements in environmental efficiency leads to reductions in air pollutant emissions, thereby enhancing air quality and positively influencing fertility. This partially supports the widely accepted notion. However, our study further argues that when environmental efficiency surpasses a certain threshold, fertility rates may decline. This results in an inverted U-shaped relationship between environmental efficiency and human reproductive health. This phenomenon is akin to the non-linear relationship between environmental pollution and economic development as described by the EKC. According to EKC theory, economic development initially leads to environmental degradation, but as economic development reaches a certain level, environmental quality begins to improve (43). Our research indicates that this reversal effect is similarly applicable to the relationship between environmental efficiency and fertility. When environmental efficiency is low, economic growth may exacerbate environmental pollution, thus negatively impacting fertility. However, as environmental efficiency increases, the positive environmental effects of economic development begin to outweigh the negative ones, ultimately contributing to higher fertility rates.

The relationship between environmental efficiency and energy demand may be a key factor underlying this non-linear link. The potential non-linear relationship between energy consumption and air quality has been widely discussed in the existing literature. On the one hand, it is widely accepted that boosting carbon emission efficiency is considered a promising means of reducing carbon emissions (28, 30, 53). It is feasible to conclude that raising environmental efficiency is a helpful approach for lowering airborne contaminants emissions and enhancing air quality by recognizing the synergy between air pollutants and CO_2_ emissions. On the other hand, increasing energy efficiency and improving environmental efficiency are strongly associated since carbon emissions and the consumption of fossil fuels are two aspects of chemical transformation. It should be highlighted that the “rebound effect”—a twofold effect on emissions—occurs when technical advancements reduce the requirement for fossil fuels, hence improving environmental efficiency. In brief, the rebound effect is the term given to describe both the immediate and the long-term consequences, such as income effects and substitution, that an emerging energy-saving innovation brings. A new technology may have a rebound impact that partially or completely cancels out any direct or immediate energy savings. The consequences of emissions thus become less predictable (54).

This conclusion represents a typical application of the EKC theory to the relationship between environmental efficiency and human reproductive health. It underscores the importance of balancing economic development with environmental protection in efforts to improve environmental efficiency. During periods of economic growth, enhancing environmental efficiency should be paired with appropriate policy interventions and technological innovations to prevent rebound effects and ensure the long-term sustainability of environmental improvements.

### 4.3 Robustness tests

Additional robustness tests were conducted to ensure the reliability of the results mentioned above.

#### 4.3.1 U-shaped relationship test

As this non-linear relationship was identified for the first time, there are no specific references available for an accurate comparison. To reconfirm the genuine existence of the inverted U-shaped curve, we conducted another test.

First, to determine whether the relationship is likely S-shaped instead of inverted U-shaped, we add a cubic factor (*X^3^*) to Equation 3 to obtain Equation 4.


(4) $$\ln TFR_{\mathrm{it}}=\beta_{0}+\beta_{1}EE_{\mathrm{it}}+\beta_{2}EE_{\mathrm{it}}^{2}+\beta_{2}EE_{\mathrm{it}}^{3}+\overset{n}{\underset{k=1}{\sum }}\theta_{k}X_{\mathrm{it}}+\mu_{i}+\delta_{t}+\varepsilon_{\mathrm{it}}$$

Second, although it is popular, using quadratic regressions to search for U-shaped connections is inaccurate. It is nearly hard to figure out whether the true functional form of a quadratic regression is quadratic, which is necessary to properly comprehend the results. Thus, the U-shape is tested in this study by applying the Robin Hood approach, which avoids presuming anything about the functional form. To explicitly test the hypothesis that the average influence of *x* on *y* changes signs at the high and low points of *x*, the Robin Hood approach establishes breakpoints for the two lines. Referring to Uri Simonsohn’s research (55), we re-estimated the relationship between variables by establishing the following Equation 5–8:


(5) $$\ln TFR_{\mathrm{it}}=\alpha +\mathrm{\beta EElo}w_{\mathrm{it}}+\mathrm{\gamma EEhig}h_{\mathrm{it}}+\sigma D_{\mathrm{it}}+\mu_{i}+\delta_{t}+\varepsilon_{\mathrm{it}}$$

within which:


(6) $$\mathrm{EElo}w_{\mathrm{it}}=\{\begin{array}{c} \begin{array}{cc} EE_{\mathrm{it}}-EE_{0}, & EE_{\mathrm{it}}<EE_{0} \end{array}\\ \begin{array}{cc} 0, & EE_{\mathrm{it}}\geq EE_{0} \end{array} \end{array}$$

(7) $$\mathrm{EEhig}h_{\mathrm{it}}=\{\begin{array}{c} \begin{array}{cc} EE_{\mathrm{it}}-EE_{0}, & EE_{\mathrm{it}}\geq EE_{0} \end{array}\\ \begin{array}{cc} 0, & EE_{\mathrm{it}}<EE_{0} \end{array} \end{array}$$

(8) $$D_{\mathrm{it}}=\{\begin{array}{c} \begin{array}{cc} 1, & EE_{\mathrm{it}}\geq EE_{0} \end{array}\\ \begin{array}{cc} 0, & EE_{\mathrm{it}}<EE_{0} \end{array} \end{array}$$

The model is an improved interrupted regression model, where *EE_0_* represents the breakpoint. As the testing method is based on a quadratic regression, the turning point of Equation 3 is chosen as the breakpoint, with *EE_0_* = 0.70. *D_it_* is the grouping variable that divides the two regions on either side of the breakpoint.

As seen in Table 3, the results of Equation 4 in column (2) show that the cubic term is not significant, thus providing stronger support for a quadratic relationship. The results of Equation 5 are located in column (3). Our priority is assessing whether there exists a relationship with an inverted U (the direction of the influence of *x* on *y* reverses after the breakpoint), which differs from the traditional interrupted regression that seeks to identify the significance of a breakpoint. Therefore, we only need to pay attention to the coefficients in front of the variables *EElow_it_* and *EEhigh_it_*. On both sides of the breakpoint, it is evident that the slopes of the two fitted lines have opposite signs and are significant within the 1% level. It suggests that the link between fertility and environmental efficiency is an inverted U.

#### 4.3.2 Replace the dependent variable

The *TFR* is more inclined to reflect women’s fertility levels, while the birth rate is a measure of fertility levels without gender characteristics. We repeat the regression adopting the crude birth rate (*CBR*) as a substitute measure of fertility levels as a way to further assess the reliability of the baseline regression model. The *WDI* database contains the statistics for the *CBR*, which is defined as the total amount of live births per 1,000 midyear population. As shown in column (4) of Table 3, the robustness of the baseline model is demonstrated by the fact that the results are basically in line with the previous results and pass the significance test.

#### 4.3.3 Variables are lagged by one period

The state of fertility is an indicator of the outcome of a pregnancy; the average gestation period for women is 37 weeks, while the production of sperm in men takes 10 weeks. Since it may be concluded that the factor 1 year before the date of birth affects human fertility, we re-estimated Equation 3 after lagging all explanatory factors by one period. Referring to column (5) of Table 3, the findings demonstrate the adjustments to the model do not alter our primary substantive conclusions.

### 4.4 Heterogeneity analysis

The impact of environmental efficiency on fertility is also contingent on the changes in the energy and industry dimensions accompanying economic development. Therefore, the influence of environmental efficiency on fertility rates varies among countries with different energy and industrial structures. Based on previous literature, we will further conduct heterogeneity analysis from the perspectives of the structure of energy consumption and industry.

#### 4.4.1 Industrial structure

Since industrial structure is a key component of the connections between human activity and the environment, it must be taken into consideration when addressing the conflict between the environment and economic growth. Sue et al. analyzed the shift in the US GDP-to-energy ratio and found that sectoral organizational reforms (structural change) are crucial for slowing down the growth of emissions (56). A panel threshold model was constructed by Zheng et al. to explore the impact of industrial transformation on air pollution (57). According to their conclusions, NO and SO pollution can be considerably decreased by lowering the GDP proportion of secondary industrial production. Additionally, cutting emissions is an advantage of a more sustainable industrial structure, which stimulates the migration of resources—like energy—from inefficient to efficient sectors.

In light of this, optimizing the industrial structure helps lower air pollution emissions and boost environmental effectiveness. It indicates that in nations with different industrial structures, the influence of environmental efficiency on fertility differs. The positive effect of environmental efficiency on fertility may be more noticeable in nations with higher proportions of energy-intensive sectors since excessively high environmental efficiency is not favorable to fertility. Thus, we propose:

*H2*: Countries with a higher proportion of energy-intensive sectors have a bigger positive impact effect of environmental efficiency on fertility.

The consumption of energy in industry is a major driver of emissions that cannot be neglected. About 25% of CO_2_ emissions from the energy sector were produced by the G7 countries (Canada, France, Germany, Italy, Japan, the United Kingdom, the United States, and the European Union) in 2020, which contribute about 40% of the global economy. The manufacturing sector is responsible for around 36% of global carbon dioxide emissions (58). Therefore, using data from the WDI, we adopt the portion of GDP that is generated by the secondary industry (*IGR*) as a proxy for the percentage of the energy-intensive sector. We categorize the countries into those with a high-emission industrial structure and those with a low-emission industrial structure by employing the grouping criterion of *IGR* median. Define a dummy variable high-emission industrial structure (*high_IGR*) and introduce the interaction term high_IGR*EE in the baseline model. The coefficient of high_IGR*EE is positive, as column (1) of Table 4 demonstrates.

**Table 4:** Heterogeneity analysis results.

	(1)	(2)
*EE*	0.413^***^	0.414^***^
	(0.0751)	(0.0698)
*EE^2^*	−0.289^***^	−0.305^***^
	(0.0633)	(0.0570)
*high_IGR*	−0.026^*^	
	(0.0137)	
EE*high_IGR	0.082^**^	
	(0.0396)	
*Ngreen_ECS*		−0.036^**^
		(0.0149)
EE*Ngreen_ECS		0.079^**^
		(0.0307)
*Control*	Yes	Yes
N	874	874
adj. *R*^2^	0.361	0.363

Standard errors in parentheses, ^*^p < 0.1, ^**^p < 0.05, ^***^p < 0.01.

The results indicate that the marginal impact of environmental efficiency is greater in countries with high-emission industrial structures, thereby validating Hypothesis H2. High-emission industrial economies, such as Belgium, Canada, and Germany, heavily rely on energy-intensive industries like steel, chemicals, and power generation (50, 59, 60). In contrast to economies with cleaner industrial structures, the green transformation of traditional industries typically brings about substantial environmental improvements. As a result, improvements in environmental efficiency have a more pronounced positive impact on alleviating fertility challenges in these countries. By transitioning to low-carbon and clean energy industries, these nations can significantly reduce carbon emissions and environmental pollution, thereby easing social pressures and economic burdens. As Abbas et al. (61) demonstrate, a 1% increase in renewable energy consumption is associated with a 2.20% rise in life expectancy and a 1.27% increase in fertility rates. This, in turn, creates better conditions for family life and childbearing, fostering a rise in fertility rates. In contrast, countries with low-emission industrial structures, such as America, Austria, and Denmark, have economies that are driven by high-tech industries, services, and clean energy. These countries have been successful in implementing robust green policies, further advancing their transition toward sustainable growth, reducing environmental costs, and enhancing environmental efficiency (44, 62). However, this also leads to diminishing marginal benefits in promoting public health through further environmental efficiency improvements, as the impact is less pronounced compared to other countries.

#### 4.4.2 Energy consumption structure

To strengthen the global economy, energy supply has been increasing. However, there are serious environmental issues associated with a significant rise in pollutant emissions as well as increasing energy consumption. In addition, distinct consumption patterns give rise to various emissions, which makes it possible for differences in the energy consumption structure (*ECS*) to lead to disparate total emissions among nations, even in cases where GDP growth is identical. Kartal et al. analyzed, with a categorical perspective on energy consumption, the possible impact of changes in energy consumption on CO_2_ emissions (63). Utilizing French data from 1970 to 2021 with a dynamic autoregressive distributed lag (DYNARDL) model, they discovered that a positive shock to coal would culminate in a significant rise in CO_2_ emissions. It suggests that the way energy is consumed will affect how numerous airborne contaminants are generated overall and that optimization of the energy structure can be effective in mitigating energy-related emissions by improving environmental efficiency while maintaining economic growth.

Combining the previous empirical findings, one can further hypothesize that different nations with varied patterns of energy consumption have particular impacts on fertility when it comes to environmental efficiency. Nations with a high-carbon ECS, where environmental efficiency is more crucial in cutting air pollution emissions, have a greater probability of having beneficial effects on fertility. Therefore, we propose:

*H3*: Fertility is positively impacted by environmental efficiency more profoundly in nations with a high-carbon ECS.

We use the percentage of coal in the utilization of energy as the measurement of ECS, and the data are collected from EIA since the optimization of ECS primarily occurs in the lowering of the ratio of high-carbon energy consumption, especially raw coal (64). Using the median of the ECS as a grouping criterion, we categorize countries into high-carbon (non-green) ECS and low-carbon (green) ECS countries. We define the dummy variable high-carbon ECS (*Ngreen_ECS*) and introduce the interaction term Ngreen_ECS*EE in the baseline model. Refer to column (2) of Table 4, where a positive coefficient on Ngreen_ECS*EE is found. It supports H3 by demonstrating that the impact of environmental efficiency on fertility is greater in nations with high-carbon ECS.

In high-carbon energy consumption countries, such as Australia, Canada, and Germany, the energy structure primarily relies on fossil fuels, and these countries generally have high levels of industrialization and urbanization. Existing studies commonly suggest that high-carbon energy consumption is associated with higher living costs and societal pressures, particularly in areas such as healthcare, education, and housing, which increases the financial burden on households and suppresses fertility intentions (61, 65). However, their higher levels of economic development and growth provide a stronger technological potential and foundation for green transformation, meaning that compared to other regions, these countries can achieve more significant environmental improvements when enhancing environmental efficiency, which is also reflected in an increase in fertility intentions. In contrast, low-carbon energy consumption countries such as Denmark, Switzerland, and the United Kingdom have actively promoted the transformation of their energy structures, reducing their reliance on fossil fuels and improving environmental quality. However, having already benefited from better social welfare systems and lower living costs, citizens’ fertility intentions have largely stabilized, meaning the effect of enhanced environmental efficiency on fertility rates is more moderate.

## 5 Moderating effect of economic development

### 5.1 Moderation effect hypothesis

The efficiency of pollutant emission levels in the process of economic growth is measured by EE in each of the models listed above. Effective EE denotes the bond between the minimal number of undesirable outputs and each unit of desirable output generated by the DMU. In the case of the same production technology, the potential reductions in pollutant emissions amount to zero (50). Therefore, the role of EE in fertility explores the impact of the environmental friendliness of unit economic growth on fertility. In reality, however, the overall quantity of emissions of pollution into the environment is dependent not only on the environmental efficiency of economic growth per unit but also on the overall amount of growth.

Fertility and economic growth have been established to be negatively correlated. However, recent studies on the inverse “J-shaped” connection that exists between economic development and fertility have revealed that under conditions of sustained economic and social development, fertility reversed in the 2000s and has since risen again in certain wealthy Western countries (14–16). There is no arguing that economic development has an impact on fertility, even though opinions on the inverse J model remain controversial. To account for the potential moderating effect of economic development, we analyze how varying degrees of economic progress might influence the impact of environmental efficiency on fertility rates. Building on this, we hypothesize the following:

*H4*: Economic development moderates the inverted U-shaped relationship between environmental efficiency and fertility, where the influence of EE on fertility differs across countries with varying levels of economic development.

We used real GDP per capita (*AGDP*) as a substitute for the degree of economic progress based on previous studies. Using statistics from the WDI database, we employed the GDP deflator to convert current prices to constant prices at purchasing power parity in 1995. We subsequently introduced the logarithm of real GDP per capita (*lnAGDP*) and the corresponding interaction factor with EE to Equation 3, which led to Equation 9:


(9) $$\begin{array}{l} \ln TFR_{\mathrm{it}}=\beta_{0}+\beta_{1}EE_{\mathrm{it}}+\beta_{2}EE_{\mathrm{it}}^{2}+\beta_{3}\ln AGDP_{\mathrm{it}}\\ \;+\beta_{4}EE_{\mathrm{it}}\ln AGDP_{\mathrm{it}}+\beta_{5}EE_{\mathrm{it}}^{2}\ln AGDP_{\mathrm{it}}\\ \;+\overset{n}{\underset{k=1}{\sum }}\theta_{k}X_{\mathrm{it}}+\mu_{i}+\delta_{t}+\varepsilon_{\mathrm{it}} \end{array}$$

### 5.2 Regression results and analysis

The corresponding coefficient for *lnAGDP* in each of Table 5’s three columns is positive and passes the significance test within the 1% level. It implies that fertility is positively impacted by the degree of economic development as a moderating variable, which appears to conflict with the findings of previous studies by the studies before (14–16). Since most OECD member nations are economically advanced, this supports the idea that their levels of development may have surpassed the turning point of the inverse J-shaped curve. As a result, they primarily reflect the positive impact of economic development on fertility, corresponding to the latter half of the inverse J-curve (16, 66). This phenomenon can be explained through the lens of family economic theory. Firstly, economic development leads to an increase in individual and family income, enhancing the ability of families to raise. Secondly, it’s necessary to recognize that economic development has a negative substitution effect on fertility. As a result of the requirement for more human capital brought about by economic growth and technological advancement, families are obligated to raise their child-rearing costs. At the same time, government financial subsidies and market-based childcare services can be applied to externalize the costs of having children (67–69). It has a favorable influence on fertility by enabling the income effect of economic growth to reconcile with the negative substitution effect in wealthy nations.

**Table 5:** The non-linear impact of EE on fertility with the per capita GDP as a moderator.

	(1)	(2)	(3)
*EE*	0.349^***^	0.322^***^	0.376^***^
	(0.0660)	(0.0727)	(0.0683)
*EE^2^*	−0.280^***^	−0.232^***^	−0.224^***^
	(0.0572)	(0.0717)	(0.0668)
*lnAGDP*	0.241^***^	0.169^***^	0.0762^***^
	(0.0188)	(0.0214)	(0.0237)
EE*lnAGDP		−0.269^***^	−0.250^***^
		(0.0349)	(0.0337)
EE^2^*lnAGDP		0.349^***^	0.276^***^
		(0.0739)	(0.0714)
*Control*	No	No	Yes
N	874	874	874
adj. *R*^2^	0.282	0.329	0.421

Standard errors in parentheses, ^*^p < 0.1, ^**^p < 0.05, ^***^p < 0.01.

The results in column (3) of Table 5 indicate that the level of economic development moderates the relationship between environmental efficiency and fertility. Specifically, the regression coefficient for the primary interaction term (EE*lnAGDP) is negative, while the coefficient for the quadratic interaction term (EE^2^*lnAGDP) is positive, both statistically significant at the 1% level. This suggests that the inverted U-shaped effect of EE on fertility weakens or flattens as economic development progresses. This finding implies that the mechanism through which environmental efficiency affects fertility may differ at various stages of economic development. Specifically, as economic development advances, improvements in income levels and education increase environmental awareness, leading to more effective implementation of environmental policies, which in turn reduces the impact of environmental efficiency on fertility (70). Furthermore, in more developed economies, changes in social structure and fertility norms contribute to the stabilization of fertility rates, further diminishing the influence of environmental efficiency on fertility (66). Therefore, economic development seems to flatten the inverted U-shaped effect of environmental efficiency on fertility, making this nonlinear relationship more moderate.

### 5.3 Further discussion

To more accurately assess the role played by the level of economic development, we conduct a test referring to a study by Haans et al. (71). By initially establishing the first-order derivative regarding *X*, we can find the inflection point X* of Equation 9 and conclude that it is dependent on the variable *Z* that acts as a moderator (as can be seen in Equation 9). We take the partial derivative concerning *Z* of the first-order equation to illustrate how the inflection point changes with the moderator variable:


(10) $$\frac{dX^{*}}{dZ}=\frac{\beta_{1}\beta_{5}-\beta_{2}\beta_{4}}{2{\left(\beta_{2}+\beta_{5}Z\right)}^{2}}$$

Since the denominator is larger than zero, the shift’s direction is determined by the value of the numerator. Specifically, a positive numerator enables the turning point to move to the right as *Z* grows, and vice versa. To explicitly test if the turning point has altered, we calculate whether Equation 10, for two values of *Z* (minimum and maximum of *lnAGDP_it_*), is substantially different from zero. This result is not significant, indicating that there is no shift in X* when the values of the moderating variable change. The threshold is 0.4188.

Following that, we analyze the curvature variation of the inverted U-shape through the coefficients of the quadratic interaction factors in Equation 9, where the relationship for the inverted U-shape flattens out when *β_5_* is positive and steepens out when the opposite is true. According to Table 5, *β_5_* is positive within the 1% significance level. It means that as the degree of growth in the economy increases, the inverted U-shaped between environmental efficiency and fertility is flattening or weakened.

It ought to be pointed out that the shape of a curve has the potential to be altered from an inverted U-shape to a U-shape by flattening. Recalling the formula for X* (the integral formula of Equation 10), we may find the precise value of *Z* at which the shape-flip takes place as X* approaches infinity (letting the denominator be 0), which is shown in Equation 11:


(11) $$Z^{*}=-\frac{\beta_{2}}{\beta_{5}}$$

The curve has variations in form as it approaches and exceeds the Z* value, but at this value, the relationship between EE and fertility is linear. With the regression outcomes from Equation 9, we calculated the above equation and observed that the value does not fall within the range of *lnAGDP* values and is smaller than the lower bound of the interval. It suggests that with the promotion of economic development levels, the curve between environmental efficiency and fertility reverses by shifting to a U-shape.

**Figure 1:**
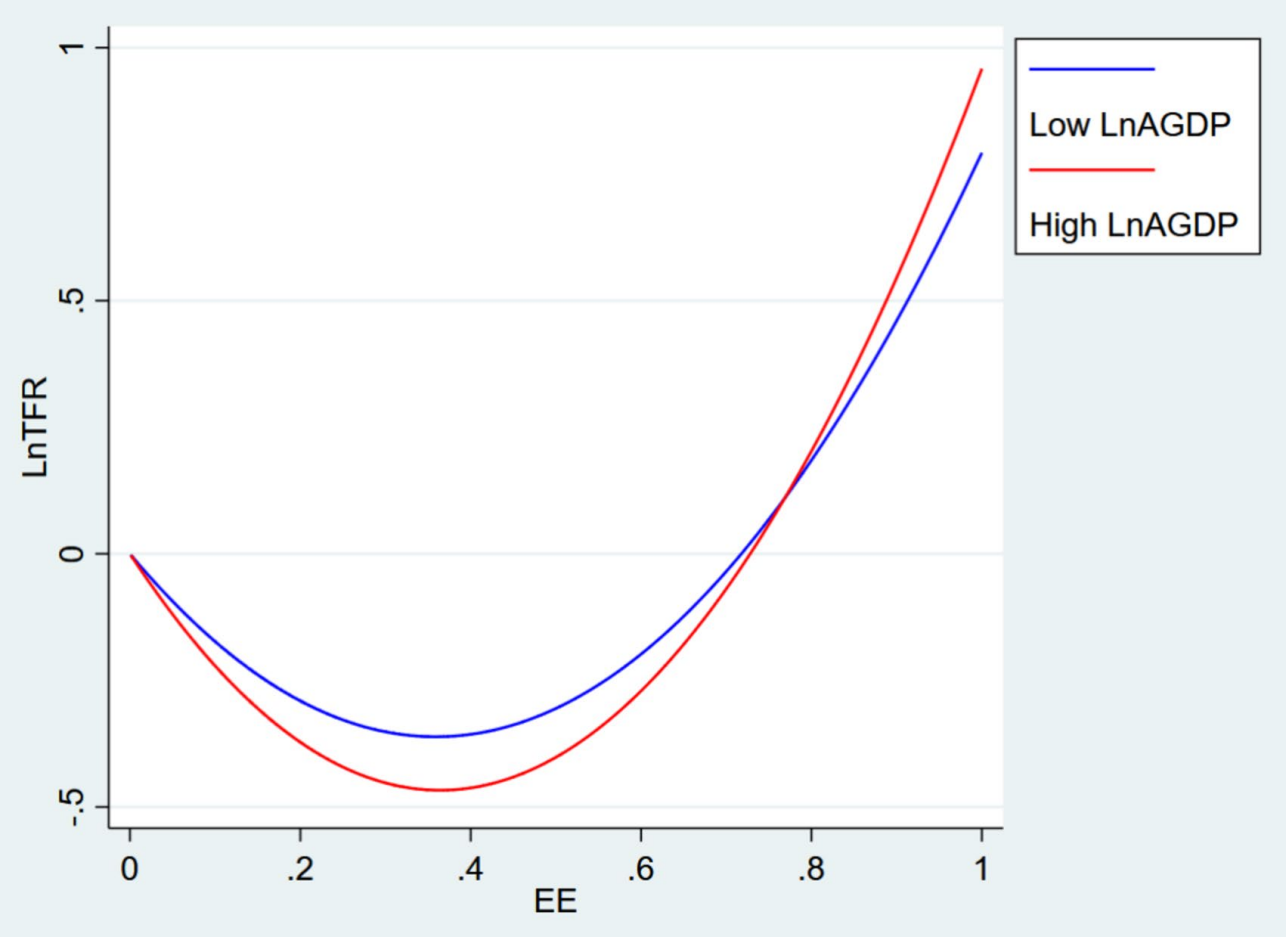
Per capita GDP as a moderator strengthens the non-linear impact of environmental efficiency on fertility.

The above findings imply that the relationship between environmental efficiency and fertility is subject to a moderating effect of economic factors. Specifically, in countries with lower levels of economic development, lower environmental efficiency has a negative impact on fertility, whereas beyond a certain threshold, improvements in environmental efficiency positively affect fertility. Figure 1 illustrates how the U-shaped curve between environmental efficiency and fertility steepens as economic growth increases. This phenomenon can be explained by the substitution relationship between economic growth and environmental efficiency regarding fertility issues. In more developed economies, improvements in environmental efficiency may be accompanied by changes in social structure, policy, and fertility norms, which together contribute to a rise in fertility rates.

Observing column (3) in Table 5, it is obvious that the primary interaction term’s coefficient is negative, while the coefficients of *EE* and *lnAGDP* have similar positive signs and are both statistically significant. It indicates that there is no effective synergy between environmental efficiency and economic development and can be analyzed in the following ways. On the one hand, according to economic theory, the EKC hypothesis suggests an inverted U-shaped relationship between economic growth and environmental degradation. Initially, as per capita income increases, environmental degradation intensifies, but after reaching a certain threshold, this relationship reverses, leading to a reduction in environmental degradation (43, 59). Considering the economic development characteristics of OECD countries, economic growth may initially lead to increased pollution, especially during industrialization and urbanization. However, with economic development and technological advancement, countries can reduce environmental pollution through environmental policies and green innovation, thereby mitigating the impact of environmental degradation on fertility fluctuation (44, 62). On the other hand, OECD countries typically have higher education levels and greater environmental awareness, with governments placing more emphasis on environmental governance. These factors help reduce the effect of environmental pollution on fertility. Meanwhile, as economic development progresses and social welfare improves, fertility rates are influenced by various factors, such as the cost of raising children and female labor force participation, which also contribute to the recovery of fertility rates (61, 65). Therefore, economic development weakens the inverted U-shaped impact of environmental efficiency on fertility by improving environmental conditions and driving shifts in social structure, and may even promote a rebound in fertility rates.

## 6 Conclusion and implications

This study examines the relationship between fertility and environmental and economic sustainability within the context of sustainable development. The results indicate an inverted U-shaped relationship between environmental efficiency (EE) and fertility, suggesting that excessively high EE may not promote fertility. A heterogeneity analysis, considering national-level industry and energy consumption structures, reveals that the positive effects of EE on fertility are stronger in countries with high-carbon energy consumption or energy-intensive sectors. Additionally, we find that economic development plays a moderating role: at lower levels of EE, economic growth negatively impacts fertility, whereas at higher EE levels, economic growth has a positive effect on fertility. These insights suggest that environmental policies focused on improving environmental efficiency can play a crucial role in enhancing both public health and fertility. Specifically, they highlight the need for targeted measures in countries with high carbon energy consumption and energy-intensive industries, where such policies can help mitigate negative impacts on fertility. Furthermore, these policies are essential for ensuring sustainable economic growth, as improving EE can positively influence fertility rates while fostering long-term environmental and social stability.

**Figure A1:**
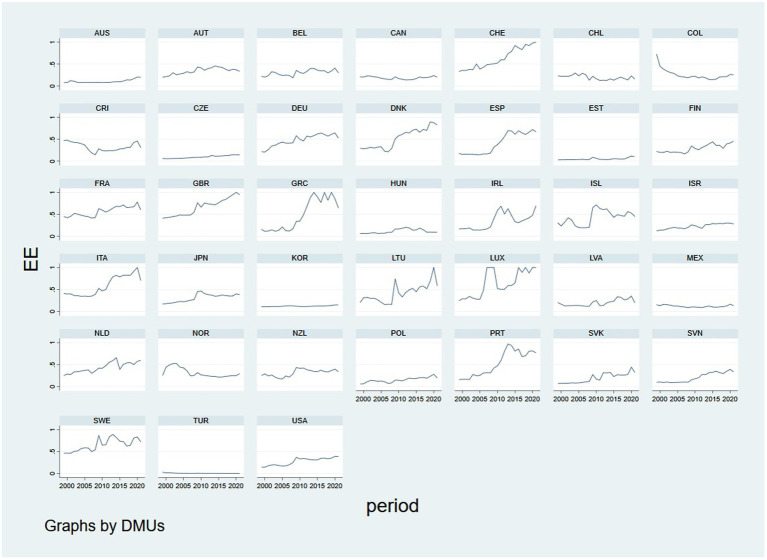
Trends in environmental efficiency in OECD countries, 1999–2021.

In this context, first, although most OECD nations have stabilized their environmental efficiency, only a few have effectively improved EE in the 21st century (see Appendix Figure A1). Countries that are lagging in improving EE should implement stricter emission reduction measures, focusing on air pollution control in industrial sectors and improving resource efficiency. The impact of EE on fertility is particularly important in nations with high energy consumption or energy-intensive industries. Policymakers should incentivize green technology innovation through fiscal measures, encouraging businesses to enhance energy efficiency in production processes. This could amplify the positive impact of EE on fertility, contributing to both environmental and public health goals.

Second, improving environmental efficiency during economic growth is crucial for the sustainable development of human society. On one hand, environmental efficiency can positively influence fertility and contribute to human sustainability only after reaching a certain threshold, which economic development helps achieve. On the other hand, increasing EE is essential for ensuring sustainable economic growth, providing a healthy environment for future generations, and offering a financially secure foundation for marriage and fertility. These efforts are integral to ensuring long-term public health and social stability.

This study has several limitations. First, due to data limitations, we use CO_2_ as a proxy for air pollution, rather than pollutants that directly affect fertility and public health. This may limit the accuracy of the environmental efficiency analysis. Future studies should include more specific air pollutants, such as PM2.5, sulfur compounds, and nitrogen oxides, which have a direct impact on human health and fertility. Second, this study focuses on the static relationship between fertility and environmental efficiency, without considering how changes in environmental efficiency over time affect public health. Future research could explore these dynamic changes, for example, by using the ML index of environmental efficiency, to better understand its long-term impact on public health and fertility. These improvements could offer deeper insights into how environmental efficiency influences both public health and fertility.

## Data Availability

The original contributions presented in the study are included in the article/supplementary material, further inquiries can be directed to the corresponding author.
